# The use of nanoparticles in the treatment of infectious diseases and cancer, dental applications and tissue regeneration: a review

**DOI:** 10.3389/fmedt.2023.1330007

**Published:** 2024-01-23

**Authors:** Ali Sobhani-Nasab, Hamid Reza Banafshe, Amir Atapour, Mahmood Khaksary Mahabady, Maryam Akbari, Abdolreza Daraei, Yaser Mansoori, Amin Moradi Hasan-Abad

**Affiliations:** ^1^ Physiology Research Center, Institute for Basic Sciences, Kashan University of Medical Sciences, Kashan, Iran; ^2^ Department of Medical Biotechnology, School of Advanced Medical Sciences and Technologies, Shiraz University of Medical Sciences, Shiraz, Iran; ^3^ Anatomical Sciences Research Center, Institute for Basic Sciences, Kashan University of Medical Sciences, Kashan, Iran; ^4^ Department of Surgery, School of Medicine, Kashan University of Medical Sciences, Kashan, Iran; ^5^ Cellular and Molecular Biology Research Center, Health Research Institute, Babol University of Medical Sciences, Babol, Iran; ^6^ Noncommunicable Diseases Research Center, Fasa University of Medical Sciences, Fasa, Iran; ^7^ Autoimmune Diseases Research Center, Shahid Beheshti Hospital, Kashan University of Medical Sciences, Kashan, Iran

**Keywords:** nanoparticle, antibacterial, anti-cancer, tissue regeneration, dentistry

## Abstract

The emergence of nanotechnology as a field of study can be traced back to the 1980s, at which point the means to artificially produce, control, and observe matter on a nanometer level was made viable. Recent advancements in technology have enabled us to extend our reach to the nanoscale, which has presented an unparalleled opportunity to directly target biomolecular interactions. As a result of these developments, there is a drive to arise intelligent nanostructures capable of overcoming the obstacles that have impeded the progress of conventional pharmacological methodologies. After four decades, the gradual amalgamation of bio- and nanotechnologies is initiating a revolution in the realm of disease detection, treatment, and monitoring, as well as unsolved medical predicaments. Although a significant portion of research in the field is still confined to laboratories, the initial application of nanotechnology as treatments, vaccines, pharmaceuticals, and diagnostic equipment has now obtained endorsement for commercialization and clinical practice. The current issue presents an overview of the latest progress in nanomedical strategies towards alleviating antibiotic resistance, diagnosing and treating cancer, addressing neurodegenerative disorders, and an array of applications, encompassing dentistry and tuberculosis treatment. The current investigation also scrutinizes the deployment of sophisticated smart nanostructured materials in fields of application such as regenerative medicine, as well as the management of targeted and sustained release of pharmaceuticals and therapeutic interventions. The aforementioned concept exhibits the potential for revolutionary advancements within the field of immunotherapy, as it introduces the utilization of implanted vaccine technology to consistently regulate and augment immune functions. Concurrently with the endeavor to attain the advantages of nanomedical intervention, it is essential to enhance the unceasing emphasis on nanotoxicological research and the regulation of nanomedications' safety. This initiative is crucial in achieving the advancement in medicine that currently lies within our reach.

## 1 Introduction

The fabrication of innovative engineered materials, especially nanomaterials, has experienced a significant surge within the past three to four decades. Emerging as versatile materials, they are utilized in diverse fields such as engineering, waste management, sports equipment, the electronic industry, optical devices, garments, food production, and cosmetic formulations, dominating virtually all sectors of daily living (1–6). The present issue focuses on an array of novel material applications in medicine facilitated through the amalgamation of nanotechnology and biotechnology. The distinctive characteristics of nanoscale materials, namely their inherent capacity for physiochemical customization and manipulation, enable the exploration of a vast array of possibilities within the medical realm. This includes the early identification of biomarkers, precise targeting of cellular and tissue components, development of sophisticated drug delivery mechanisms, accurate staging and evaluation of medical conditions, and treatment of degenerative ailments. Such capabilities have profound implications for medical advancements and innovations. Engineered nanomaterials have been precisely characterized as possessing one dimension measuring less than 100 nm (7, 8).

In the field of medicine, the definition of a drug is characterized by a degree of flexibility, wherein it may encompass a diverse range of formulations such as a nano drug comprising particles measuring 200 nm or greater in size. Moreover, the terminology “nanoparticle” possesses a comprehensive connotation, encompassing not only spherically-shaped organic and inorganic nanomaterials but also cuboidal, star-shaped, needle-like, spheroidal or intricately-structured forms possessing complex geometries, with an aerodynamic diameter of less than 100 nm. Certain articles expound upon the fundamental characteristics of particles within the respective scope of the subject matter, whilst varying articles do not prioritize such discourse. The objective of this disclosure is to ensure that the audience comprehends the broadest interpretation of this term, as it is utilized in the subsequent articles (9–12).

### 1.1 Nanoparticle agonist bacterial infection

The issue of anti-microbial resistance poses a worldwide challenge that is currently impacting contemporary healthcare systems. The emergence of antimicrobial resistance across various classes of antibiotics can be attributed to the suboptimal prescribing patterns of antibiotics (13). Undoubtedly, this phenomenon will considerably influence the future effectiveness and utilization of antibiotics in the realms of community and hospital care on a worldwide scale (14, 15). The World Health Organization (WHO) unveiled its inaugural list of antibiotic-resistant pathogens in February of 2017, detailing a dire need for the prompt development of novel anti-microbial therapies (16, 17). Among the twelve pathogens exhibiting resistance, it was observed that seven strains evinced resistance to beta-lactam antibiotics. The three pathogens classified as “Critical” exhibit resistance towards carbapenems specifically imipenem, while four other pathogens are resistant to fluoroquinolones, such as ciprofloxacin, which are extensively employed in the clinical setting. The aforementioned fact is a source of concern as it portends challenges not only for prescribing practices but also regarding the acquisition of appropriate antibiotics for patient treatment in the future. Despite the current situation, the World Health Organization (WHO) has expressed that this presents a favorable circumstance for the research and development (R&D) industry to innovate novel antibiotics, thereby establishing a novel target for forthcoming research tactics. Bacteria, which are classified as prokaryotes due to their lack of a nuclear membrane, are categorized as either Gram-positive or Gram-negative based on the structure of their cell wall. A Gram stain is a widely used laboratory test for classifying bacteria based on the capacity of the bacterial cell wall to absorb and hold onto crystal violet dye. Gram positive and Gram negative bacteria differ in the thickness of their peptidoglycan layers in their cell membranes. As a result, while Gram negative bacteria lose their crystal violet stain during the decolorization process, Gram positive bacteria retain it (18). Gram-positive bacteria are characterized by the presence of a rigid cell wall comprised of a thick layer of peptidoglycan. This peptidoglycan is composed of carbohydrate polymers that are cross-linked by peptide residues (19). Teichoic acid is detected on the outer surface of Gram-positive bacteria, which endows them with the capacity to sequester metal ions and function as a safeguard system against the immune response mounted by the host organism (20). Lipoteichoic acids are detected in the cellular membrane, facilitating surface adherence. In contrast, Gram-negative bacteria possess a peptidoglycan layer that is thinner and more inflexible, featuring substantially reduced levels of cross-linking. This layer is enveloped by a lipid membrane that displays lipopolysaccharides (LPS) on its surface (21). Methicillin-resistant S. aureus (MRSA) is of particular clinical importance due to its resistance to multiple antibiotics. Staphylococcus aureus, a Gram-positive bacterium, exemplifies unique resistance to various antibiotics, with Methicillin-resistant MRSA serving as a notable instance of clinical significance. The layperson frequently associates MRSA with its antibiotic resistance. MRSA infections necessitate extended therapy regimens, frequently involving potent antibiotics, and consequently result in heightened occurrences of patient hospitalization and public expenditure. Multiple approaches seek to employ nanotechnology as a means of addressing this problem (22). Many strategies aim to use nanotechnology to tackle this issue.

**Figure 1:**
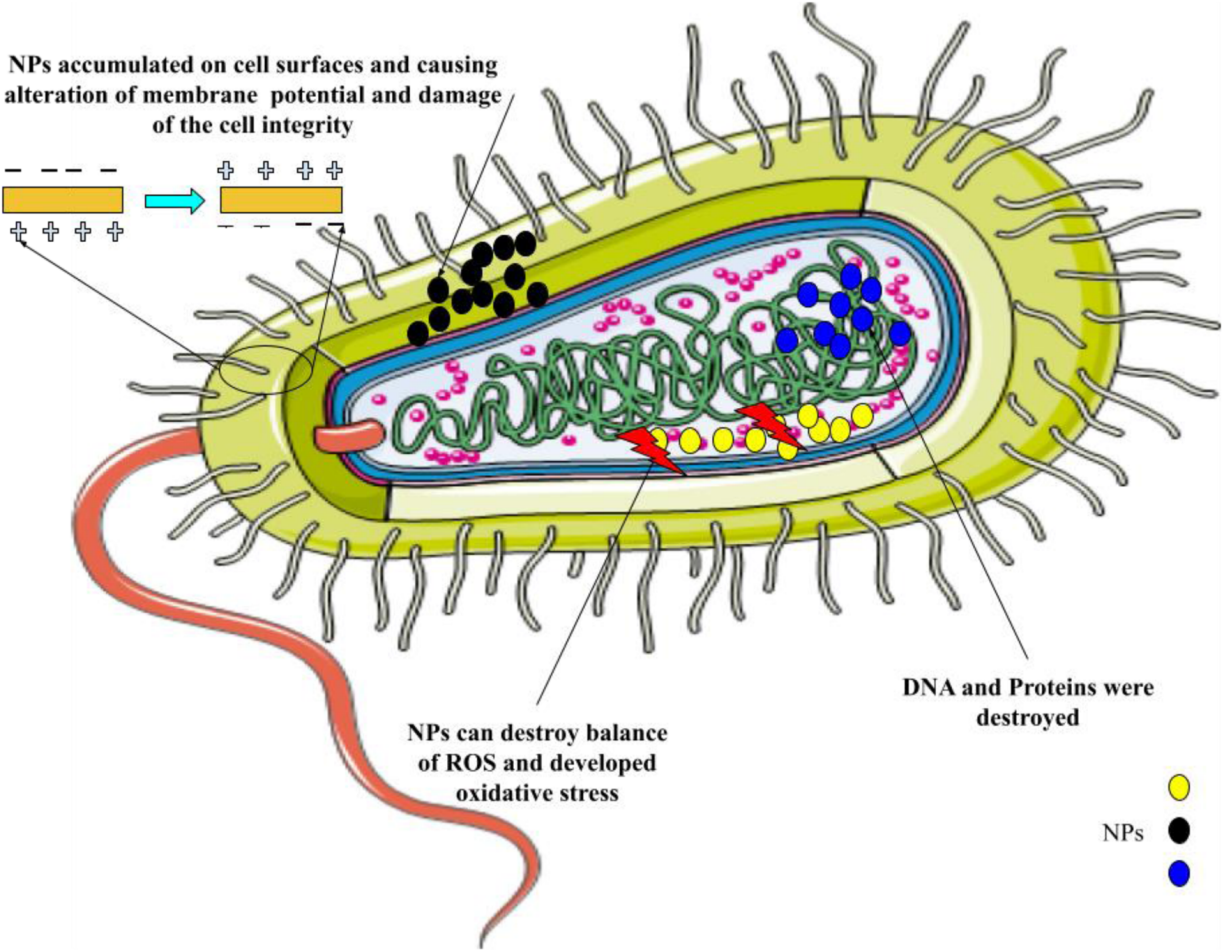
The primary mechanisms of nanoparticles’ antimicrobial activity are as follows: breaking down the pathogen cell wall, which increases permeability; producing reactive oxygen species (ROS), which upsets redox homeostasis and damages cellular structures; and attaching to intracellular structures and molecules, like DNA and protein, which causes their dysfunction.

The employment of conventional oral or intravenous pharmaceuticals to manage microbial infections is linked with a host of difficulties. The current treatment protocols, characterized by the administration of substantial doses as a strategy to guarantee the delivery of adequate quantities to the intended microbial targets, exhibit drawbacks such as inadequate efficacy and the potential for adverse reactions, culminating in the evolution of drug resistance amongst the targeted microorganisms. The insufficiency of unconventional therapeutic modalities and tactics to surmount the aforementioned issue has engendered considerable apprehension among governmental organizations, medical experts, and ultimately, the global populace owing to its conspicuous influence on public health. One viable strategy for combating this issue involves the utilization of nanomaterials to augment and potentiate the antimicrobial effectiveness of both established and innovative medicinal interventions (23–26). Through mechanisms such as disrupting the membrane potential and integrity of bacterial cells, preventing the formation of biofilms and ROS production, strengthening host immune responses, and blocking RNA and protein synthesis by inducing intracellular processes, these NPs primarily reduce the resistance properties of bacteria (Figure 1). Table 1 mentions several studies conducted in this field.

**Table 1:** Nanoparticles against multidrug resistant (MDR) bacteria: mechanisms and characteristics.

Nanoparticles	Size (nm)	Bacteria-targeted & antibiotic resistant	Anti-bacterial mechanisms	Antimicrobial activity/toxicity factors	References
Au	1–100	Methicillin-resistant Staphylococcus aureus (MRSA)	Bacterial membrane and wall injury, disruption of the respiratory chain, decreased ATPase, tRNA decline, membrane potential loss	Size of particles and roughness	(27–31)
Ag	1–100	A. baumannii, Pseudomonas Aeruginosa, MRSA, vancomycin-resistant, extended-spectrum betalactamase (ESBL)-producing organisms, MDR Escherichia coli,, Klebsiella pneumoniae, carbapenemand polymyxin B-resistant, Staphylococcus epidermidis, carbapenemresistant P. aeruginosa, Enterococcus (VRE) and carbapenem-resistant Enterobacteriaceae (CRE)	ROS production, bacterial membrane disintegration, cytochrome inhibition, lipid peroxidation, proton gradient dissipation, increased membrane permeability, cell wall synthesis inhibition, adhesion to the cell surface, ribosome destabilization, and DNA base intercalation are all adverse effects of oxidative stress.	Shape of particles and particle size	(28–32)
Cu	2–350	MDR E. coli, A. baumannii	Degradation of proteins and DNA, production of reactive oxygen species, lipid peroxidation, and loss of cell membrane potential.	Size and particle amount	(28, 30–33)
Si	20–400	MRSA	ROS-induced cell wall disruption	Shape, stability and particle size	(28, 31)
Al	10–100	E. coli	ROS-induced cell wall disruption	–	(29, 30)
Fe_2_O_3_	1–100		ROS induces oxidative stress, which consists of O^−2^, singlet oxygen, OH, and H_2_O_2_.	Strong chemical activity, tendency to aggregate, oxidation by air leading to magnetism loss and dispersibility.	(29, 31)
ZnO	10–100	K. pneumonia, Enterobacter aerogenes, E. coli, ESBL-producing E, K. p, MRSA,. coli, Klebsiella oxytoca	The production of ROS, membrane disruption, adhesion to the surface of the neutropenia cell, as well as lipid and protein damage	Concentration and Particle size	(29, 30, 32, 34)
MgO	15–100	E. coli and S. aureus	ROS production, lipid peroxidation, electrostatic interaction, and alkaline impact	pH, concentration, and Particle size	(29)

#### 1.1.1 Nanoparticles for the therapy of tuberculosis

Tuberculosis (TB) could be a zoonotic and anthropozoonotic infection with a complex pathogenesis, created by microbes from Mycobacterium tuberculosis complex (MtbC), primarily M. tuberculosis, and in a lesser sum by the contaminations with other mycobacteria such as M. bovis, M. canetti, M. caprae, M. africanum, and sometimes M. microti or Mycobacterium pinnipedii (35, 36). The present resurgence of TB at a regional and global level is impeded by factors such as the emergence of multidrug-resistant strains, intercurrent immunosuppressive conditions, the high costs and low production outputs of recently endorsed antitubercular antibiotics, and moderately effective vaccination measures. As a result, these factors have hindered progress toward eradicating TB. TB is a global infectious disease that affects over one-third of the human population. Despite recent progress in treatment and prevention, the latest report by the World Health Organization identifies it as the primary cause of infectious-bacterial deaths amongst adults, with a staggering 10 million new cases and 1.5 million deaths attributed to TB in 2018 alone. Furthermore, it has been observed that TB serves as the primary contributing factor to hospital fatalities in certain regions that are characterized by a high prevalence of the disease (37).

**Figure 2:**
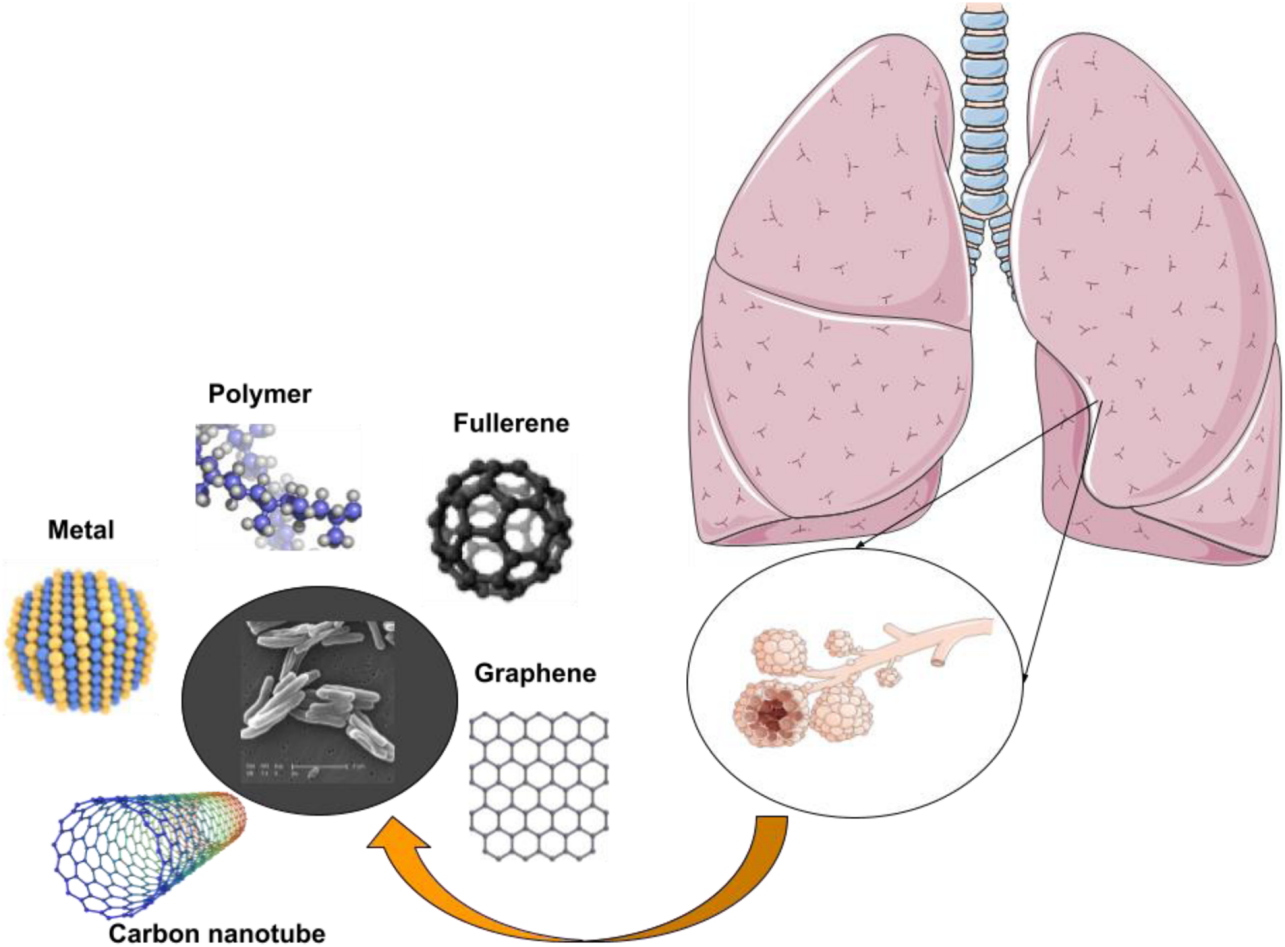
Nanotechnology is being used to develop medicine delivery systems that specifically target alveolar macrophages and granulomas for the treatment of TB.

Nanotechnology and nanoparticle science providing innovative approaches and new-practical solutions for several critical-issues, including TB (37–39). Extended treatment durations and frequently changing drug dosages pose significant obstacles to the effectiveness of current TB medicines, since they frequently result in patients not adhering to prescribed regimens or receiving inadequate care. Regarding extensively drug-resistant (XDR) tuberculosis (DRT) and multidrug-resistant (MDR) TB, the primary contributing factor to the illness's recurrence is the patient's noncompliance. Medical researchers are faced with a difficulty since MDR-TB and XDR-TB are growing increasingly prevalent in developing countries and pose a significant danger to world health (40). Novel approaches to drug delivery that leveraged ligands and nanocarriers were investigated, and a summary of several nano delivery systems was provided. It is now quite helpful to employ pulmonary nanodrug delivery systems as a therapeutic agent to treat tuberculosis (Figure 2). The advantages of employing nanomaterials to deliver medications for the treatment of infectious lung disorders include targeted drug delivery, enhanced drug solubility, and decreased toxicity, fewer adverse effects compared to standard drug regimens that produce MDR and XDR, and synergistic therapeutic effects. Table 2 mentions a number of studies conducted in this field.

**Table 2:** The application of nanoparticles in the diagnosis and treatment of tuberculosis.

Type of nanoparticle	Drug delivery/diagnosis	Target	Therapeutic agent/diagnostic agent	Mode of action	References
Au, PLGA-PEG-SAPEG-PLGA	Drug delivery	Tuberculosis cells	Rifampicin	Chemotherapy method	(41)
Au	Diagnosis	Tuberculosis 38 antigen	Antibody	SPR (Surface plasmon resonance)	(42)
Au	Diagnosis	Tuberculosis DNA	DNA	SPR (Surface plasmon resonance)	(43)
Au-SMVLD	Treatment	Tuberculosis cells	SMVLD	Bacteriostatic	(44)
SPIO	Diagnosis	Tuberculosis cells	Anti- Tuberculosis antibody	Magnetoresistive biosensor	(45)
SPIO, Au	Diagnosis	CFP10	Antibody	Immunoassay	(46, 47)
SPIO, Au, CdS & Carbon quantum dots	Diagnosis	IFN-γ, TNF-α IL-12,	Antibody	Immunoassay	(48)
SPIO	Diagnosis	Tuberculosis cells	Antibody	Contrasting agent	(49)
SPIO	Drug delivery	Tuberculosis cells	Isoniazid	Chemotherapy method	(50)
Silica, beta-glucan	Drug delivery	Tuberculosis cells	Isoniazid	Chemotherapy method	(51)
PLG	Drug delivery	Tuberculosis cells	Isoniazid	Chemotherapy method	(52)
Isoniazid, rifampicin, pyrazinamide	Drug delivery	Tuberculosis cells	Rifampicin	Chemotherapy method	(53–56)
Dendrimer	Drug delivery	Tuberculosis cells	Rifampicin	Chemotherapy method	(57, 58)
Chitosan	Drug delivery	Tuberculosis cells	Rifampicin	Chemotherapy method	(59)
Aptamer	Diagnosis	Ag85A	Apt22	Flow cytometry	(60)

### 1.2 Nanoparticle vaccines against infectious diseases

**Figure 3:**
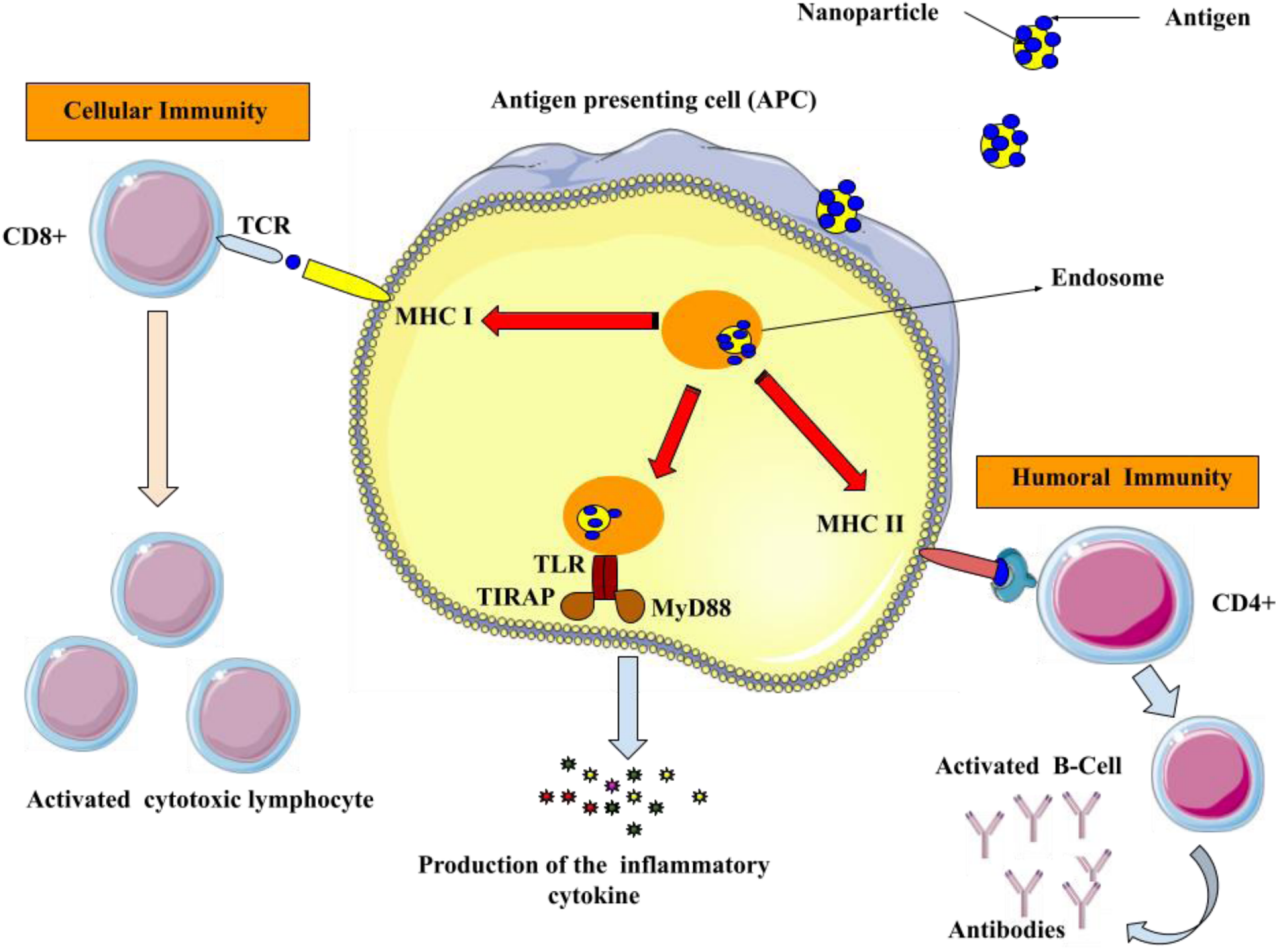
Utilizing surface modified nanoparticles to deliver antigenic compounds specifically to APCs. Antigens that are produced inside the body are shown together with class I major histocompatibility complex (MHC I) on the surface of APCs to CD8^+^ T lymphocytes. Upon the interaction of MHC I and T-cell receptor (TCR) in the presence of co-stimulatory molecules and cytokines, the activated CD8^+^ cells initiate cytotoxicity to eliminate the infected cells. Furthermore, the antigens are displayed on the surface of the APCs through class II major histocompatibility complex (MHC II) molecules to activate the helper (CD4^+^) T cells. Afterward, CD4^+^ cells stimulate B-cells to generate antimicrobial antibodies.

Vaccination is a medical intervention that involves the administration of an antigenic substance into an individual's body to elicit an immune response and establish adaptive immunity against a targeted pathogen (61, 62). The efficacy and cost-efficiency of applying preventive measures to contain infectious diseases have been convincingly demonstrated. Numerous consequential maladies such as tetanus, mumps, measles, smallpox, polio, rubella, pertussis, yellow fever, and diphtheria have been effectively eliminated or effectively controlled by means of vaccinations (63, 64). Despite the notable achievements of vaccination therapies, numerous disease entities remain devoid of an effective prophylactic tactic. Examples of such ailments include acquired immunodeficiency syndrome (AIDS), tuberculosis, malaria, and dengue fever. Consequently, there is a continual pursuit of novel vaccination formulations and technologies (65). Vaccine formulations typically comprise attenuated subunit protein antigens and inactivated microorganisms that elicit a specific immunological response. Each system possesses distinct advantages and disadvantages, and there is often a trade-off between safety and efficacy (66, 67). Antigen might be encapsulated in the core of the nanoparticles or attached to their surface. Antibodies, Fab-fragments, peptides, and other targeting molecules can be used to decorate the surface of nanoparticles, which can enhance their distribution into antigen-presenting cells (APCs) and trigger both innate and adaptive immune responses (Figure 3) (68, 69).

The capacity of NPs to regulate immune responses toward attaining intended outcomes is imperative in the development of vaccines leveraging nanotechnology. NPs hold the potential as a dual-purpose agent in augmenting and intensifying protective immunity by serving as both a delivery vehicle and an immune-stimulatory adjuvant (70, 71). Nano vaccines, which utilize NPs as carriers or adjuvants, offer several distinct advantages over conventional vaccines. These advantages include the ability to decrease the rate of antigenic degradation, enhance the stability of antigens, immunogenicity and improve vaccine therapeutic efficacy, facilitate efficient phagocytosis and rapid processing by, enhance cellular membrane penetrability and APCs (72). The current research indicates that nanocarriers, including liposomes, dendrimers, and virosomes, exhibit properties that enhance cytokine induction and antibody response. As a result, recent efforts have been directed toward the development of vaccine delivery strategies employing these nanocarriers (73). The mentioned nanocarriers represent a diverse group of nanomaterials, renowned for their distinctive structural designs, suitable for serving as potential paradigms for drug delivery modalities. Furthermore, they enhance the bioavailability of compounds, offer stabilization and safeguarding of more delicate agents such as proteins, reduce the occurrence of adverse effects, and facilitate active targeting (74). Table 3 mentions several studies conducted in this field.

**Table 3:** Application of different nanoparticles against viral infections.

Virus	Nanocarrier platforms	Constituents	Route of administration	Experimental outcomes	Advantage	References
HIV	Peptide-based nanofibrous hydrogel	DNA, Methylamino group, Naphthalene acetate, Tyrosine, Phenylalanine, and Glycine	Subcutaneous (SC), Intradermal (ID), and Intramuscular (IM)	Significantly elevated levels of IFN- and IL-4 cytokine were detected.	Biological compatibility and bioassurance	(75)
MCV	MCV-like particles	Polyomavirus capsid proteins, VP1 (Viral proteins 1)	IM	The seroprevalence of anti-MCV antibodies was found to be substantially elevated in mice immunized with MCV VLPs. Cross-reactive antibodies against LPV VLPs and BKV VLPs were found to have a low seroprevalence, accounting for 4.4% and 2.6% of the reactivity against MCV VLPs, respectively.	Biodegradable, biocompatible, and Nontoxic	(76)
HSV	Polymeric NPs	Poly (lactic-co glycolic acid)	Mucosal	In response to exposure to the attenuated viral antigen, the inoculated fish generate anti-VHSV immunoglobulin (Ig), activating the humoral immune response components. In immunized groups, the percentage of anti-VHSV inhibition was substantially higher than in unvaccinated challenged groups.	Simple to manufacture, low toxicity, nonimmunogenic, and biodegradable	(77)
Influenza A virus	AuNPs	Cytosineguanine-rich oligonucleotide, AuNPs	Intranasal	While M2e was coupled to AuNPs, anti-M2e serum Ig ratios were observed to increase.	Enhanced bioavailability and half-life, reduced toxicity, and biocompatibility	(78)
Hepatitis B	Polymeric NPs	(Poly lactic-coglycolic acid) PLGA, (Poly-lactic acid) PLA	IM and pulmonary	Anti-HBsAg antibody concentrations in PLA and PLGA were significantly higher than in ordinary HBsAg.	Targeted drug delivery that is site-specific, biodegradable and nonimmunogenic, and has minimal toxic effects.	(79)
Viral infections	Nanogel	Cationic alginate-poly ethylenimine	Intraperitoneal	Nanogels significantly increased anti-OVA IgG1 production, but had little effect on IgG2a and IgG2b expression. Nanogels enhanced IgG isotypes and anti-OVA IgG in a dose-dependent manner and increased OVA-specific IFN- by 60-fold.	The achievement of nonimmunogenic, exceptionally biocompatible, controlled and prolonged drug delivery	(80)
HPV (Human papillomavirus)	VLPs (Virus-like particles)	VLPs, L1, and L2 proteins	–	–	Specific to the target site, environmentally friendly, and nontoxic	(81)

### 1.3 Nanoparticles in dentistry

**Figure 4:**
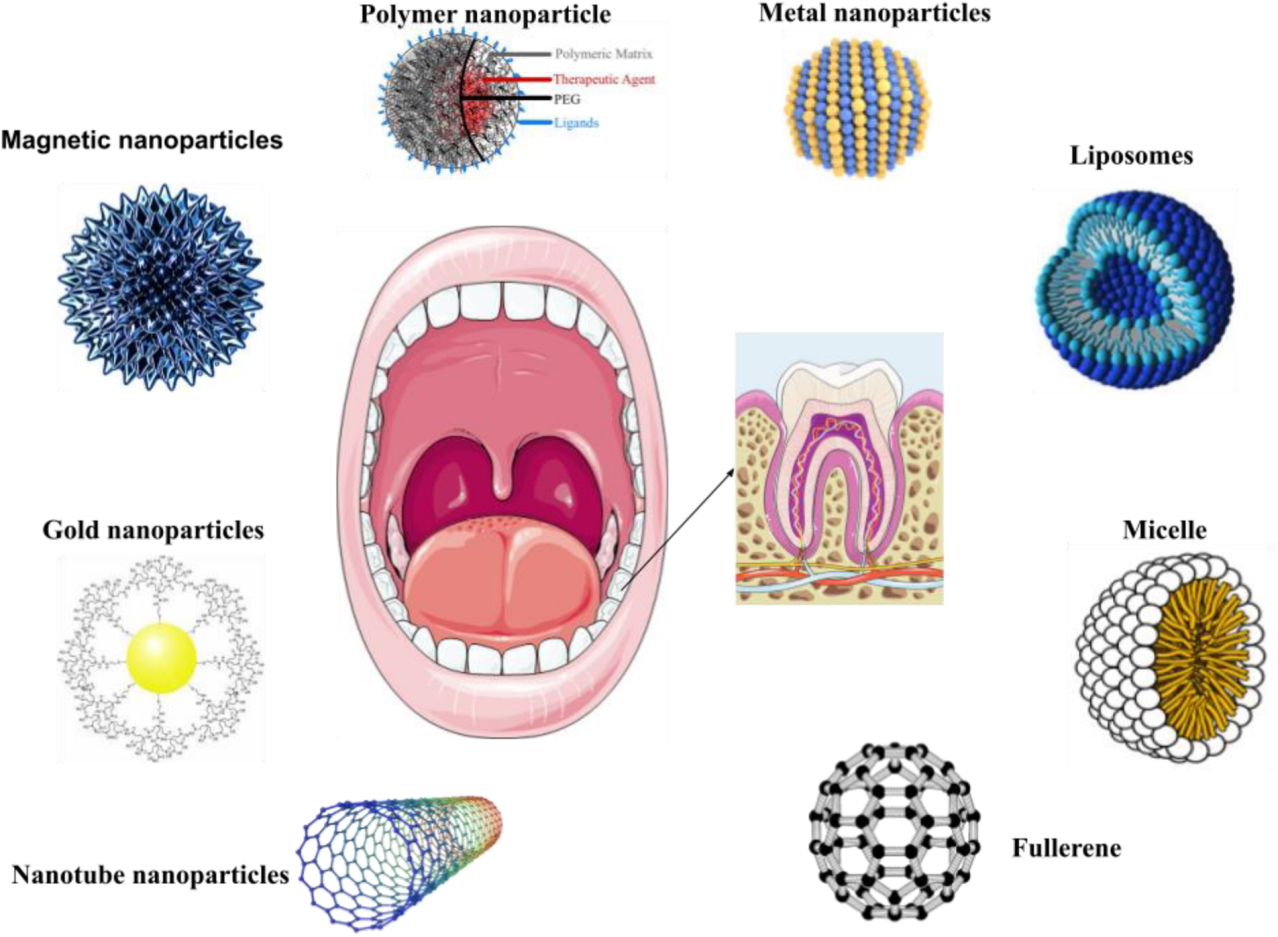
Utilizations of organic and inorganic NPs in dental care and treatment. NPs are utilized in dental inventions and diagnostic procedures. NPs are utilized in the development of oral disease preventative medications, prostheses, and dental implants. Nanomaterials have the ability to provide oral fluids or medications, effectively preventing and treating certain oral diseases such as oral cancer, while significantly promoting oral healthcare.

NPs are naturally occurring entities that are ubiquitous in the environment and hold significant utility in various daily applications. The advancement of nanotechnology has led to a sharp rise in the use of NPs in cosmetics. Better absorption of substances through the skin, longer-lasting effects, and increased stability are just a few of the benefits that come with using NPs. Currently, sunscreen products use NPs as UV filters most frequently in cosmetics. Zinc oxide (ZnO) or Titanium dioxide (TiO_2_) particles are frequently utilized as ultraviolet filters or as additives in toothpaste, with a focus on titanium dioxide or silicates in toothpaste formulations. NPs are ubiquitously found in numerous edibles, nutritional additions, and spritzers utilized for veneering, disinfecting, and saturating (82–84). They possess the ability to enhance, for instance, the preservation of the edibility and integrity of the comestible, its flavor profile, and its physical coherence. In certain jurisdictions, silicon dioxide, magnesium oxide, and titanium dioxide are subject to rigorous evaluation and authorization as permissible food additives (85). In dentistry, NPs have gained growing importance as deliberate inclusions in various products (Figure 4). This materials enhance the fundamental traits of resin-based composites, such as their capacity for high polish ability and retention of gloss stability (86). In addition to their conventional applications, these materials hold potential value as constituents of scaffolding frameworks used for tissue engineering purposes (87).

Dental materials that are designed to intentionally release NPs are relatively infrequent, such as those utilized in occlusion indicator foils and scanning sprays for computer-aided design or computer-aided manufacturing (CAD/CAM). Conversely, nanoparticles may be generated as by-products during milling procedures employed for filler production (88). Numerous dental materials, including but not limited to resin-based composites, cement, and impression materials, are known to comprise fillers. Consequently, it has been projected that nanoparticles are extant in roughly 3,500 dental materials. Nanotechnology exhibits considerable potential for numerous applications in everyday life. Diverse scientific entities at the global level, including both research collectives and national/international agencies, have invested significant efforts in the development of this innovative and auspicious technology (89, 90). Table 4 mentions several studies conducted in this field.

**Table 4:** The application of nanoparticles in the field of dentistry.

Nanoparticles	Applications in dentistry	Advantages	Toxicity and adverse effects	References
Carbon	Nanotubes dental filling, a protective layer applied to the surface of teeth.	A significant surface area facilitates the delivery of active substances to viable cells, while also exhibiting strong adhesion to the tooth surfaces, as well as the surfaces of dentin and cementum.	The reactivity of carbon nanotubes (CNTs) is significantly influenced by factors such as their structure, size, surface characteristics, and purity. Under certain circumstances, nanotubes have the ability to elicit inflammatory and fibrotic responses by traversing membrane barriers.	(91, 92)
Graphene	The application of a dental coating that is appropriate for implantation purposes, with the aim of reducing biofilm formation.	The attributes of cost-effectiveness, fracture resistance, and low density are associated with the formation of a homogeneous crystal lattice, as well as the potential for treating bacterial biofilm.	The toxicity of graphene is contingent upon its specific characteristics, including its structure, size, and oxidative state. The processing procedures involved in post-synthesis can introduce metallic contaminants, which have the potential to elicit varying levels of toxicological reactions.	(93, 94)
Hydroxy apatite (HAp)	Dental hypersensitivity can be decreased by substances that possess the ability to serve as cavity fillers. Additionally, these substances can postpone the process of auxiliary demineralization and facilitate the healing of enamel surfaces.	The nano-sized hydroxyapatite (HAp) particles exhibit a high degree of integration into the tooth tubules. The composition exhibits similarities to teeth and bone, displaying biocompatibility and the ability to be adsorbed onto the enamel surface of teeth. Its primary function is to provide protection to the teeth by forming a synthetic enamel film, thereby addressing periodontal deficiencies.	Protein-particle complexes can form as a result of their binding with proteins, and afterwards undergo elimination by macrophages within tissues. The aforementioned particles were transported and distributed within the body's circulatory system, ultimately reaching and dispersing within the pulmonary system, spleen, and hepatic system. The toxicity of NPs has the potential to impact the inflammatory response, signaling system, and oxidative stress.	(95, 96)
Zirconia	The substance has the ability to decrease the attachment of bacteria to the surface of the tooth, thus offering a safeguard against dental caries. Additionally, it serves as an efficient agent for polishing.	Teeth exhibit comparable mechanical qualities and coloration, possess minimal cytotoxicity, demonstrate favorable biocompatibility, and display notable resistance to breakage.	Zirconium oxide NPs have been found to potentially provide both short-term and long-term dangers. For instance, exposure to these nanomaterials has been associated with considerable DNA damage in human T-cells and the induction of apoptosis, as well as the reduction of cell proliferation in human mesothelioma and mouse fibroblast cell lines. Additionally, it was shown that this phenomenon has the ability to initiate cellular oxidative stress, ultimately resulting in cell death. Research findings have demonstrated that these NPs possess the ability to halt the progression of the cell cycle, in addition to their capacity to traverse different physiological barriers, hence leading to detrimental consequences.	(97–99)
Silica	The dental filling agent is utilized for tooth polishing and serves as a preventive measure against dental cavities. Additionally, it possesses antibacterial properties and is employed in the treatment of dental hypersensitivity.	Biocompatible materials provide a low toxicological impact, have low density, and demonstrate notable adsorption capabilities. Furthermore, these materials are particularly advantageous due to their cost-effectiveness. When utilized as a polishing agent, it has been shown that the use of this substance leads to a decrease in the roughness of the tooth surface.	The toxicological consequences are contingent upon the method of entry and the physiochemical properties. Recent research studies have demonstrated that silica NPs have the potential to generate silicosis, similar to crystalline particles, and can also contribute to the development of lung cancer. Silica nanoparticles (SiNPs) elicit cytotoxic effects. In addition, it has been observed that this phenomenon can also lead to the occurrence of oxidative stress and trigger apoptosis, with the extent of these effects being influenced by the size and dosage of the substance. Several studies have also documented the genotoxic effects of SiNPs, including DNA damage and modulation of genes involved in apoptosis and autophagy. Additionally, SiNPs have been found to have immunotoxic effects.	(100–102)
Titania	Mainly dental implants	The long-term impact on dental implants is influenced by surface modification, which offers several benefits such as reduced bacterial adherence and enhanced hardness.	Fine particles (FPs) exhibit less toxicity compared to non-particulate pollutants (NPs). The substance is introduced into the body via the process of inhaling. Epidemiological research have indicated that individuals employed in TiO_2_ producing facilities are afflicted with cancer. Titanium dioxide nanoparticles (TiO_2_ NPs) have the ability to accumulate within the brain via traversing the blood-brain barrier, specifically in the cortex and hippocampus regions. Exposure to TiO2 induces activation of microglia, formation of reactive oxygen species (ROS), and activation of signaling pathways that contribute to cellular apoptosis and inflammation.	(103–105)
Silver	Antimicrobial drugs are utilized in the field of dentistry to combat microbial infections. Dental restorative materials are employed to restore the structure and function of damaged teeth. Dental prosthetics refer to artificial devices used to replace missing teeth or oral structures. Dental implants are specifically designed to serve as permanent fixtures within the oral cavity.	It is well-documented that the substance in question has the ability to reduce bacterial colonization and promote oral health. This efficacy is attributed to its reduced molecular size, which facilitates its penetration through bacterial membranes. The material exhibits biocompatibility, with minimal toxicity and prolonged antibacterial efficacy.	Silver nanoparticles (AgNPs) have been found to elicit harmful effects. Prolonged exposure to silver has the potential to induce a medical condition known as argyria. The adverse impacts of silver nanoparticles (AgNPs) can be attributed to the generation of reactive oxygen species (ROS). Both silver ions and silver nanoparticles (AgNPs) are implicated in the manifestation of toxicity. Silver nanoparticles (Ag NPs) are implicated in the generation of oxidative stress and genotoxicity, activation of lysosomal acid phosphatase (AcP), disruption of actin, stimulation of hemocyte phagocytosis, and inhibition of Na-K-ATPase.	(106, 107)

### 1.4 Nanoparticles for cancer therapy

**Figure 5:**
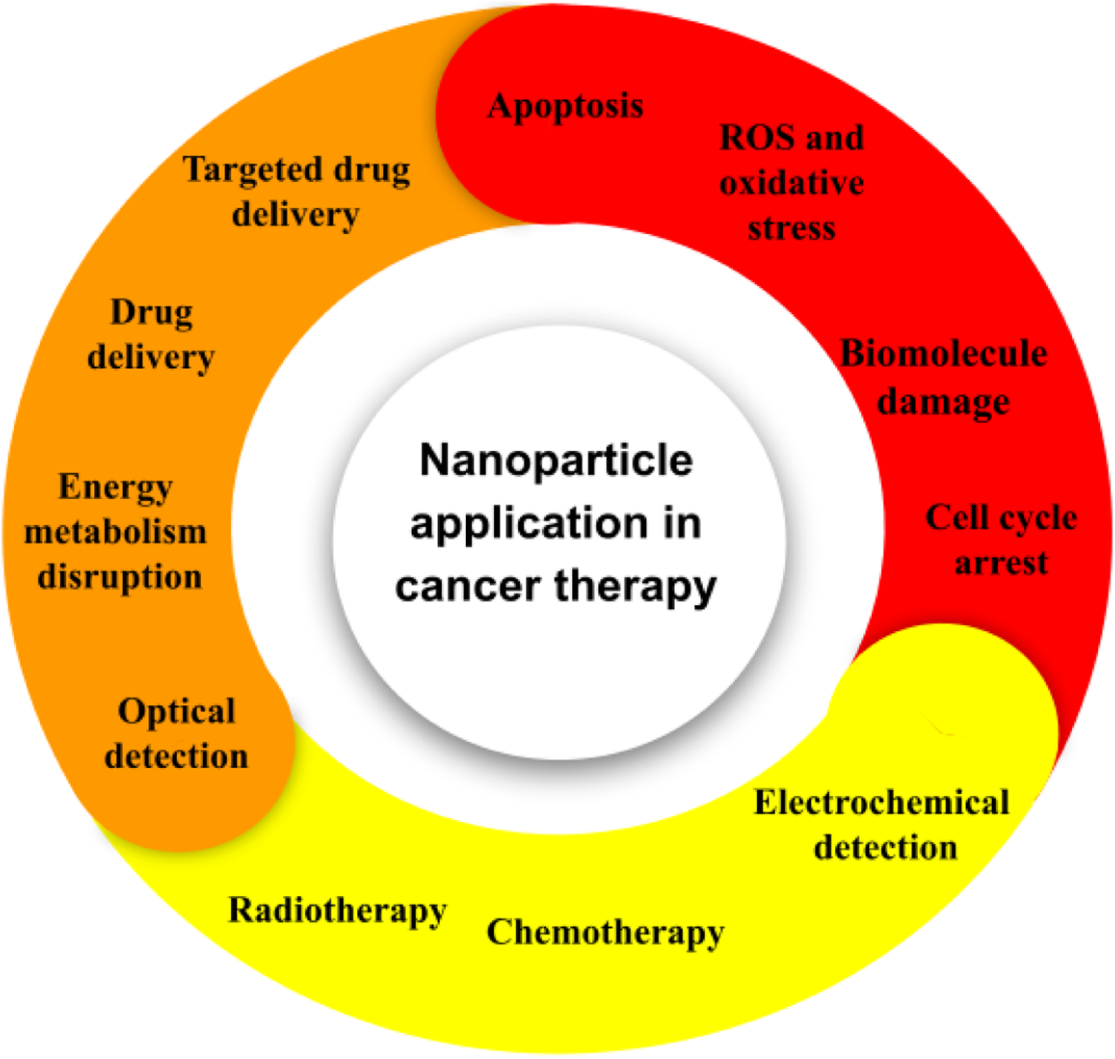
The application of nanoparticles in the field of cancer. At present, several platforms are under investigation for their potential use in both cancer therapy and diagnosis.

Cancer denotes a comprehensive class of ailments that are typified by the unregulated proliferation of cells and their invasiveness into surrounding tissues (108, 109). Considerable endeavors spanning multiple years have been devoted to identifying diverse risk elements associated with cancer. The etiology of certain cancers has been notably linked to particular acquired factors within the environment, including radiation and pollution. Adopting an unhealthy lifestyle, marked by bad dietary habits, consumption of tobacco products, smoking, stress, and absence of physical activity, profoundly influences the determination of cancer risk (110, 111). Although external factors have been widely acknowledged as significant contributors to carcinogenesis, the precise impact of somatic mutations in proto-oncogenes, alterations in tumor suppressor gene expression, and variations in the genes involved in DNA repair mechanisms remain a challenging proposition to assess accurately. A mere 5%-10% of cancer instances correlate with genetic inheritance (112). The progression of chronological age is deemed a pivotal determinant for the onset of cancer and its diverse manifestations. The traditional therapeutic modalities employed in the management of cancer consist of several methods which include surgical intervention, radiation therapy, chemotherapy, targeted therapy, hormone therapy and immunotherapy (113, 114). Radiation therapy and Chemotherapy exhibit cytostatic and cytotoxic capabilities (115). Frequently associated with severe adverse reactions and a significantly elevated chance of relapse, these methodologies constitute a notable concern within the medical community. The administration of this agent is commonly associated with the occurrence of suppression of bone marrow, neuropathies, skin disorders, and gastrointestinal, alopecia, and fatigue as the prevailing manifestations of treatment-related adverse effects. Moreover, the administration of certain drugs may entail unique adverse effects, such as anthracyclines and bleomycin-induced cardiotoxicity and pulmonary toxicity. The progress in precision medicine has been bolstered by the emergence of targeted therapy. Despite the advances in therapeutic interventions, numerous inescapable detrimental repercussions, such as the emergence of multi-drug resistance, continue to impede the effectiveness of the treatment regimens (116, 117). Immunotherapeutic agents have exhibited auspicious outcomes not only in the treatment of primary cancer but also in their ability to prevent distant metastasis and diminish the frequency of recurrence (118). Notwithstanding, immunotherapy constitutes a significant factor precipitating autoimmune diseases. Moreover, scholarly investigations and empirical findings posit that immunotherapy exhibits a comparatively lower efficacy in addressing solid tumors in contrast to lymphoma (119). The cancers under consideration manifest an anomalous extracellular matrix (ECM) that presents a formidable hurdle for the infiltration of immune cells (120). This statement suggests that recent developments in targeted therapies and immunotherapies have introduced interventions that disrupt vital signaling pathways implicated in both malignant and homeostatic properties within the epidermis and dermis. As a consequence, the implementation of these therapies may lead to adverse dermatologic events (dAEs) (121). About the aforementioned particulars, there has been an upsurge in the need for the development of innovative approaches toward attaining accurate cancer therapy in recent times. Contemporary undertakings have been initiated to tackle the constraints of prevailing therapeutic methodologies by employing nanoparticles (Figure 5) (122–129). Nanoparticle-mediated drug delivery systems have been observed to offer advantages in the treatment and control of cancer by exhibiting favorable pharmacokinetics, accurate targeting, diminished adverse effects, and mitigated drug resistance (130, 131).

Following the recent strides in the field of nanotechnology, numerous nanotherapeutic medications have been successfully commercialized and extensively promoted. Furthermore, an abundance of these drugs has advanced to clinical trials since 2010. Advances in the field of drug delivery systems and combatting multi-drug resistance (MDR) in the context of tumorigenesis have been achieved through the development of nanotherapeutic drugs. Such drugs have facilitated the pursuit of combination therapy and effectively inhibited resistance mechanisms associated with drug treatments (132). In the 1960s, an initial endeavor was taken to implement the use of nanotechnology within the field of medicine, specifically at the esteemed academic institution of ETH Zurich, it is a Swiss public research university located in Zürich (133). This amalgamation has demonstrated enhanced efficacy in the development of diverse diagnostic equipment and superior therapies. NPs have been documented to possess a significant ability to penetrate deep tissues, thereby promoting the enhancement of the permeability and retention (EPR) phenomenon. Moreover, it should be noted that the surface characteristics of a substance exert a significant influence on its bioavailability and half-life, primarily by facilitating its passage across epithelial fenestrations (134). An instance of this phenomenon is observed in the use of NPs that have undergone coating with polyethylene glycol (PEG), a hydrophilic polymer, which generates a decreased tendency for opsonization and also manages to evade clearance by the immune system (135). Furthermore, the modularity of the release kinetics of therapeutic substances or active components can be achieved through the manipulation of particle polymer attributes. Collectively, the unique characteristics exhibited by nanoparticles play a pivotal role in modulating their therapeutic efficacy in the management and treatment of cancer. Table 5 mentions several studies conducted in this field.

**Table 5:** Nanoparticles and cancer cell death mechanisms.

Nanoparticles	Cell lines	Mechanism of action	References
DNA-modified magnetic	MCF-7	The inhibition of RNA marker expression	(136)
Au, Ag	hPBMCs	Cytokine production and complement activation	(137)
Gold NP-tagged toxin	MCF-7	Reduced expression of CDK-4 and MAPK	(138)
Au@ZIF-8	EMT-6	ROS production	(139)
Fe_3_O_4_@AuNC@erlotinib	PANC-1	Targeting selectively excessively expressed EGFR	(140)
Iron oxide was functionalized with GOx and PDA	4T1, MCF-10A and MDA-MB-231	Photothermal treatment and ROS-mediated injury	(141)
V_2_O_5_	B16F10, A549, and PANC1	ROS-dependent apoptosis	(142)
Fe_3_O_4_	HepG2	RAS signaling through ATP-citrate lyase	(143)
Fe@Fe_3_O_4_@heparin	4T1, HUVEC cell	ROS production	(144)
PEGylated rhodium nanodots	CT-26	Reduced levels of TNF- and IL-6	(145)
Au	B16	Increased expression of Caspase 3, Caspase 9, Bid, and Bax, and decreased expression of BCl2.	(146)
Au NPs-PEG-RNase A conjugate	SW-480	ROS production	(147)
Au	B16 F10	Apoptosis mediated by the mitochondrial path	(148)
RBC membrane-coated PLGA	Pancreatic ductal adenocarcinoma	Modification of tumor microenvironment	(149)
PEGylated ZnO	PANC1	ROS-dependent apoptosis	(150)
ZnO	THP-1	Mitochondrial membrane degradation and increased reactive oxygen species	(151)
Ag	HeLa	SubG1 arrest and apoptosis/necrosis of cells	(122, 149)
Pt	A549	Apoptosis induction and cell cycle arrest	(152)
TiO_2_	LL2	Oxidative stress as well as cytokine activation	(153)
MoS_2_ nanoflakes	MDA-MB-231	Selective ROS synthesis and photothermal treatment	(154)
Pt	Human foreskin fibroblast cell	DNA damage and DNA replication inhibition	(155)
CeO_2_	Mouse fibrosarcoma cell line	ROS-dependent apoptosis	(156)
CeO_2_	A549	ROS-dependent apoptosis	(157)
ZnO	MCF-7	Increased expression of caspase-8 and p53	(158)
TiO_2_	HepG2, IMR-90, MCF-7 and A549	Oxidative stress	(159)

### 1.5 Nanoparticle approaches in neurodegenerative diseases

The brain assumes the mantle of being the most intricate organ in the human anatomy. The entity in question is intricately involved in the regulation of various cognitive, behavioral, and emotional activities. The organ in question is susceptible to various disorders and afflictions, ranging from physical trauma to malignancies and cognitive degenerative conditions. Neurological disorders are the primary contributors to impairment and a significant factor in mortality. Neurodegenerative disorders pose a significant risk to human welfare. The prevalence of age-dependent disorders has escalated, attributable to the rise in elderly populations witnessed in recent times. Prominent cases of neurodegenerative illnesses include Alzheimer's disease, Parkinson's disease, Huntington's disease, amyotrophic lateral sclerosis, frontotemporal dementia, and spinocerebellar ataxias. Many illnesses exhibit varying pathophysiological mechanisms; some result in cognitive dysfunction and memory impairments, while others disrupt an individual's motor, communication, and respiratory functions (160, 161). Numerous medications, which have demonstrated promise in enhancing cerebral architecture and operation in animal models, encounter a plethora of difficulties such as distribution, selectivity, and toxicity. For a considerable duration, researchers have encountered the formidable obstacles of formulating pharmaceutical substances capable of penetrating the physiological impediment of the blood-brain barrier, as well as navigating the electrical and chemical defenses of the brain, while simultaneously achieving targeted localization with minimal deleterious consequences. In recent times, nanotechnology has surfaced as a crucial methodology for the alteration and manipulation of diverse entities at the atomic scale to achieve targeted properties. The utilization of this particular methodology has demonstrated its efficacy in both diagnosis and treatment of cerebral diseases and disorders by improving drug delivery and enhancing their effectiveness. Given the present significance and ongoing advancements in research, technology may greatly improve healthcare systems by providing user-friendly and highly effective diagnosis and treatment approaches (162, 163). Table 6 mentions several studies conducted in this field.

**Table 6:** The utilization of nanotechnology in the context of central nervous system (CNS) conditions.

Nano-materials	Drug delivery	Disease	Advantages	Disadvantages	References
PRP CsiRNA-RVG-9r-liposomes	Prp CsiRNA RVG-9rPrp Cs	NPMD (Neurodegenerative protein misfolding diseases)	Optimize the efficacy of medication delivery, prolong the drug's presence in the bloodstream, and expedite its ability to cross the blood-brain barrier.	Research conducted on animals has demonstrated that the introduction of nanoparticles has the potential to induce type III severe allergic reactions in mice.	(164)
Fus-liposomes-rhFGF20	rhFGF20	Parkinson's disease	The objective is to optimize the permeability of BBB, extend the duration of therapeutic effects, boost the effectiveness of encapsulation, create formulations with controlled release properties, and enhance the biological activity of medicines.	Not mentioned.	(164)
RVG29-liposomes	N-3,4-Bis(pivaloyloxy)-dopamine	Parkinson's disease	The activation of the substantia nigra, the ability to effectively pass the blood-brain barrier, and the sustained presence of drug concentration.	Not mentioned.	(165)
PEG-liposomes-MBs	GDNF+Nurr1	Parkinson's disease	This technology has several advantages, including a slow release mechanism, a prolonged half-life, and the ability to facilitate ultrasound-guided blood-brain barrier passage.	The BBB exhibits limited permeability.	(166)
RMP7-lf-PEG-liposomes	Quercetin	Alzheimer's disease	This treatment possesses several notable characteristics, including a high BBB entrance rate, selectivity towards SK-N-MC cells, and a sustained release of the drug.	The activation of an inflammatory response can potentially occur.	(167)
NGF-SM-ApoE-liposomes	Nerve growth factor, surface serotonin modulator, ApoE	Alzheimer's disease	The extended release of nerve growth factor (NGF), its potent bioactivity, significant blood-brain barrier (BBB) permeability, binding affinity to A1–42 and SK–N-MC cells, and the efficacy of NGF.	–	(168)
Empty Cell	Nanomicellar system (SANS)	L-DOPA	The extended duration of drug release, straightforward production process, and transparency of the epidermis render this formulation very suitable for topical use.	The absence of targeted approaches.	(169)
The micelles formed by Pluronic P85/F68.	Baicalein	Parkinson's disease	This formulation exhibits the potential to achieve sustained release, enhanced blood-brain barrier permeability, and increased cellular concentration due to its inherent self-forming and stable characteristics.	Impairment of blood-brain barrier (BBB) permeability has the potential to cause harm to the structure and content of mitochondria.	(170)
Empty cell	Mixed-shell polymeric micelle (MSPM)	None	The addressing of A*β* deposition is motivated by several key factors, including the higher affinity of some compounds, their ability to prevent the generation of pathogenic factors, their excellent penetration of the blood-brain barrier (BBB), and their high level of biocompatibility.	Extended metabolic duration.	(171)
C(NCAM-C3)T(TPP)- M(micelle)N(nano)	Resveratrol	Alzheimer's disease	The suppression of proinflammatory factors can be achieved through the utilization of an efficient manufacturing method, enhanced blood-brain barrier penetration, and a high level of encapsulation efficacy.	A long metabolic time.	(172)
Empty cell	Micelle curcumin	Amyloidogenesis model	The phenomenon of spontaneous production, high encapsulation efficiency, sustained drug release, and blood-brain barrier penetration.	It has not been targeted.	(173)
PAMAM dendrimers	Carbamazepine	Neurodegeneration	The objective is to improve the fluidity of the packages, boost the durability of the product, achieve superior drug packaging attributes, reduce the peripheral and cellular cytotoxicity of the packaging, and significantly enhance the bioavailability.	The substance exhibits limited tolerance to acid and alkali environments, higher concentrations result in enhanced lethality, and its long-term efficacy in drug sustainability is diminished.	(174)
G4HisMal-dendrimers	Boc-L-histidine	Alzheimer's disease	The blood brain barrier penetration rate is enhanced, resulting in superior biocompatibility.	Not mentioned.	(175)
Lactoferrin-coupled PAMAM dendrimers (PAMAM-lf)	Memantine	Alzheimer's disease	The characteristics under consideration include sustained drug release, regulated drug release, prolonged drug half-life, effective encapsulation capacity, high drug delivery efficacy, efficient blood-brain barrier penetration, and targeted drug delivery to the brain.	It is possible that the substance may result in hematotoxicity.	(176)
Phosphorus dendrimers	Phosphorus	Parkinson's disease	The compound has anti-HIV activity and demonstrates the capacity to suppress fibrosis associated with -SYN.	Hepatotoxicity.	(177, 178)
Carbosilane dendrimers	None	Parkinson's disease	The reduction of reactive oxygen species (ROS) has been shown to have a neuroprotective effect by preserving neuronal cells and inhibiting the fibrillation of amyloid-beta peptide (ASN).	Not mentioned.	(179)
Dopamine-loaded PLGA nanomaterials	Dopamine	Parkinson's disease	The process of absorption can be decelerated, resulting in reduced rates of uptake, while yet exhibiting minimal levels of toxicity. The presence of these medications in the bloodstream, coupled with their high biocompatibility and low reactive oxygen species (ROS) levels, renders them very suitable for prolonged utilization.	Incite an inflammatory reaction where it is needed.	(180)
Collagen-coated PLGA	None	Parkinson's disease	It has good biocompatibility, excellent cell adhesion, and some potential to promote cell growth.	Not mentioned.	(181)
PLK2-PLGA-NP	PLK2	Parkinson's disease	The formulation's exceptional biocompatibility and superior encapsulation abilities are only two of the qualities that make it the best choice for long-term drug delivery.	Not mentioned.	(182)
Fe_3_O_4_-PEG/PLGA-OX26	Magnetic Fe_3_O_4_ nanoparticles, OX26	Alzheimer's disease	This medication may have strong drug loading, magnetic targeting, biocompatibility, extended release, and regulated release.	Larger particles.	(183)
Hollow gold nanoparticles	Xanthoceraside	Neurodegeneration	The achievement of detecting high drug loading and improving fluidity is attainable.	No targeted ability	(184)
Hollow au/Ag nanostars	None	Neurodegeneration	Characterized by an increased surface area, robust Raman reactivity, and notable sensitivity to near-infrared radiation.	Unknown biological toxicity.	(185)
Concave cubic Qu-P80-AuPd	Quercetin	Alzheimer's disease	The observed characteristics of the substance include low cytotoxicity, high permeability across the blood-brain barrier, localisation within lysosomes, and exceptional biocompatibility.	Not mentioned.	(186)
GNRs-APH-scFv, GAS	Thermophilic acylpeptide hydrolase	Alzheimer's disease	The photothermal impact is robust, exhibiting a high sensitivity to infrared light, while also demonstrating minimal toxicity and maintaining stable physical and chemical features.	The degree of self-BBB penetration exhibits a slightly inferior performance..	(187)
The screen-printed electrodes were modified with single-wall carbon nanotubes and gold nanoparticles.	None	Parkinson's disease	The key attributes encompassing optimal sensitivity, substantial stability, minimal harm, and instantaneous monitoring.	Not mentioned.	(188)
The utilization of functionalized random networks comprising carbon nanotube (CNT) structures.-CNT	None	Parkinson's disease	Increased sensitivity, enhanced detection accuracy, and optimal integration of DOPA.	Not mentioned.	(189)
Carbon nanotubes (CNTs)	None	Parkinson's disease	The biocompatibility of the material is favorable, as it exhibits a reduced proliferation of glial cells while promoting an enhanced proliferation of stem cells.	Not mentioned.	(190)
SWCNT-PEGs-lf	L -1 6-hydro-xydopamine	Parkinson's disease	The key attributes of interest include precise striatal targeting, exceptional biocompatibility, effective blood-brain barrier penetration, substantial drug loading capability, negligible toxicity, and sustained release.	It has the potential to elicit a specific inflammatory reaction.	(191)
EMT nanomaterials	None	Alzheimer's disease	The suppression of fibrinogen interactions in atypical blood clots.	Not mentioned.	(192)
SBA-15 (silica holed nanorod)	L-DOPA	Parkinson's disease	The compound has a notable rate of penetration through the blood-brain barrier (BBB), demonstrates substantial drug loading capacity, and displays exceptional biocompatibility.	No targeted ability.	(193)

### 1.6 Nanoparticles in tissue repair and regeneration

The practice of tissue and organ transplantation has been hindered by numerous challenges, including limited access to donors, the requirement for immunosuppression, as well as low success rates due to the rejection of the transplanted material. Consequently, the field of tissue engineering and regenerative medicine (TERM) has recently been experiencing a significant rise in interest as an alternative solution. This multidisciplinary field continues to rapidly expand. The interdisciplinary fields of biology, materials science, and engineering have been synthesized to facilitate the production and design of synthetic structures that mimic natural tissues and organs. These structures are not limited to implantable devices, but can also include miniature, modeled versions of the aforementioned organs (194). Achieving a biomimetic extracellular matrix (ECM) composition in a tissue's three-dimensional (3D) scaffold for cells that is endowed with suitable mechanical strength, facile monitoring of cellular activities, and provision of bioactive agents, necessitates a nanoscale methodology over a macroscopic one, for optimal performance. NPs have the potential to offer an efficacious means of regulating scaffolds' properties, including precise manipulation of their mechanical strength and the provision of controlled bioactive agent delivery (195, 196). Furthermore, several disadvantages and constraints, namely low solubility, unstable bioactivity, and truncated circulation half-life of bioactive molecules (e.g., growth factors, cytokines, inhibitors, genes, drugs, etc.), as well as contrast agents, have positioned NPs as among the most appropriate alternatives for the delivery and monitoring of bioactive agents in various applications (124, 197).

The ramifications of nanotechnology have resulted in a fundamental transformation of conventional and rudimentary modalities in TERM towards more intricate and productive mechanisms. In the realm of tissue engineering and regenerative medicine, nanoscale products, including nanofibers and nanopatterned surfaces, have been employed to influence cellular behavior alongside NPs. The employment of concurrent therapeutic and imaging mechanisms, incorporation of unconventional biomaterials possessing enhanced spatiotemporal management within scaffolds, manipulation of the discharge of diverse bioactive agents—notably growth factors—to govern the trajectory of stem cells and morphogenesis, regulation of the mechanical potency of scaffolds for hard tissue utilization, and reduction of toxicity and improvement of biocompatibility via tissue-targeted administration constitute a range of potential uses for NPs in TERM (198, 199). NPs can be developed utilizing a diverse range of materials, including ceramics, metals, and both natural and synthetic polymers. Nanostructured materials have emerged as highly favored candidates in tissue engineering and TERM due to their advantageous attributes, such as elevated penetration capability, amplified surface area with customizable surface properties, and compositional variability. These properties render them highly effective for a range of applications in TERM, including imaging, strength reinforcement, bioink, antimicrobial activity, and bioactive agent carrier functions (200). Table 7 mentions several studies conducted in this field.

**Table 7:** Nanoparticles and their uses, particularly for skin regeneration and rejuvenation.

Nanoparticle	Description	Function/use	References
Silver and gold	Sizes ranging from 1.1 to 1.6 nm	In vivo skin healing in rat models. This study aims to investigate methods to increase cell proliferation *in vitro* and promote full-thickness wound healing.	(201, 202)
Gold	Biosynthesized gold nanoparticles (AuNPs) exhibit a high degree of biocompatibility and are associated with a reduced incidence of adverse effects.	The process of granulation tissue production. The topic of discussion pertains to the activity of antimicrobial agents. The properties of skin regeneration. Incorporating a capacity to diminish the appearance of wrinkles. Enhance skin lightening, facilitate skin healing, exhibit a cleansing action, diminish inflammation and reactive oxygen species (ROS) levels, and decelerate collagen depletion and the breakdown of elastin.	(203–205)
Silver	–	This study aims to investigate the potential effects of certain interventions on keratocyte and fibroblast proliferation, modulation of the innate immune system, wound healing pace, and the rate of scarring. The topic of discussion pertains to the activity of antimicrobial agents.	(206–208)
Nanoceria	3–5 nm-sized spherical cerium oxide	Low doses have the ability to reverse the effects of UVA-induced photo toxicity, migration, and proliferation.	(209, 210)
Copper (Cu and CuS)	The particles under consideration have diameters of 20, 40, and 80 nanometers, respectively, and exhibit a spherical morphology.	The promotion of endothelial cell migration and proliferation, which is dependent on size and dose, facilitates the accelerated healing of full-thickness skin wounds. In vitro, there was an observed elevation in the expression of collagen 1A1, along with a concurrent augmentation in the production of neovascularization in rat models.	(211–213)
Zinc ferrite (ZnFe_2_O_4_)	–	The antimicrobial action is achieved through the utilization of several pathways.	(214, 215)
Silver sulfadiazine	–	The antimicrobial action, namely targeting biofilms, is of particular interest.	(216, 217)

### 1.7 Toxicology of nanoparticles

The successful implementation of nanomedicine and attainment of its medical efficacy hinges upon the comprehension of the toxicity about nanomaterials. Nanostructures demonstrate significant prospects in the field of medicine due to their capacity to exhibit chemical and biological activity, as well as their capability to access areas that traditional techniques are unable to reach. Specifically, nanostructures can be administered via inhalation, ingestion, or translocation through the skin, and once within the body, can permeate tissues, cells, and physical barriers. This allows for the potential to traverse across biological barriers, such as the blood-brain barrier, and to reach vital organs. Nanostructures still carry the risk of unintentional bodily injury, regardless of any potential benefits. Over the course of twenty years, the field of nanotoxicology has demonstrated that the intricate interactions between nanomaterials and cellular, animal, human, and environmental systems are exceedingly intricate (218). These entities have been associated with a variety of detrimental health effects, including cellular apoptosis, inflammation, exacerbation of asthmatic symptoms, fibrosis, chronic lung diseases marked by persistent inflammation, and carcinogenic processes. Significantly, the aforementioned toxicological investigations have underscored the imperative need to shun certain physical and functional characteristics of artificially designed nanomaterials. The conscientious application of responsible research and innovation in the realm of nanomedicine is legitimately anchored in the paramountcy accorded to nanotoxicity and nanotoxicology as its focal points of inquiry. The field of nanotoxicology is one that experts, regulatory agencies, and researchers can work together to investigate. Investigating this interdisciplinary area provides a means for different stakeholders to work together, which will aid in the ongoing discussions about the proper regulation and safe use of NPs (219, 220). Table 8 mentions several studies conducted in this field.

**Table 8:** *In vitro* and *in vivo* research on the gene toxicity of nanoparticles.

Nanoparticles	Toxic effects	References	*In vitro* research	References	*In vivo* research	References
SWCNTs (Single-wall carbon nanotubes)	The presence of inflammation within the olfactory bulb. This phenomenon facilitates the ROS, augments oxidative stress levels, and hampers cellular growth and apoptosis.	(221, 222)	Chinese hamster fibroblasts (V79 cell line) were exposed to single- and double-strand DNA lesions for a duration of 3 h at a concentration of 96 µg/cm^2^.	(223)	The present study investigates the genotoxic effects resulting from inhalation exposure in mice of the C57BL strain. The phenomenon under consideration elicits an expeditious inflammatory response, fibrotic tissue formation, heightened oxidative stress, and cellular hyperplasia.	(224)
MWCNTs (Multiwall carbon nanotubes)	The presence of inflammation within the olfactory bulb. This phenomenon facilitates the generation of ROS, augments oxidative stress, and hampers cellular growth and apoptosis.	(221, 222)	The objective is to trigger apoptosis in the stem cells of mouse embryos by activating the P53 protein, hence inducing DNA damage.	(225–227)	The lack of genotoxicity shown in rats The induction of DNA damage has been observed in the lung cells, bone marrow, and leucocytes of mice.	(228–231)
Silver	The findings of this study indicate that there is an elevation in oxidative stress levels and a reduction in the antioxidation capacity of antioxidative enzymes in the frontal cortex and hippocampal regions of mice.	(232)	The mutagenic and genotoxic effects of AgNPs were established by doing the micronucleus assay and comet assay.	(233–237)	The size-dependent toxicity of DNS destruction is shown in mice lung cells and testis.	(238)
Gold	The application of this technology proves to be advantageous in the fields of gene and medication delivery, as well as deep tissue imaging.		Gold nanoparticles (AuNPs) exhibited chromosomal aberrations, micronuclei production, oxidative DNA damage, and strand lesions when exposed to different cell lines.	(239–242)	The genotoxic effects of gold nanoparticles (Au NPs) were shown to be negative in mice. However, when rats were exposed to chronic and acute intraperitoneal administration of 10 and 30 nm Au NPs, DNA damage was observed in the liver, blood, and cerebral cortex cells, as assessed by the comet assay.	(243)
Titanium dioxide (TiO_2_, nanotubes and nanocomposite	The observed effects include the induction of oxidative stress, neuroinflammation, genotoxicity, dysregulation of neurotransmitters, disruption of signaling cascades, and alterations in synaptic plasticity.ts	(244, 245)	The genotoxicity of 20 nm TiO_2_ nanoparticles (NPs) was seen in Syrian hamster embryo fibroblasts over a range of doses (0.5–10 mg/cm^2^). This genotoxicity was attributed to the generation of reactive oxygen species (ROS) following the contact of the NPs with the cell membrane. A study has documented the presence of a DNA lesion that is dependent on the dosage when HEpG-2 cells are exposed to varying concentrations (10–100 µg/ml) of TiO_2_ nanoparticles. The comet and micronucleus assays have revealed the occurrence of dose-dependent micronuclei formation and DNA strand breaking in human cells.	(236, 246, 247)	The genotoxic effects of titanium dioxide (TiO_2_) particles have been observed in mice following oral exposure for a duration of 5 days. Furthermore, exposure to TiO_2_ particles during fetal development has been associated with DNA deletions.	(247)
Magnetite (Fe_2_O_3_)	The adverse effects of iron oxide nanoparticle exposure have been documented to include neuroinflammation, apoptosis, and infiltration of immune cells.	(248–250)	The application of 5.1 and 10.2 µg/cm^2^ doses of the substance resulted in the manifestation of dose-dependent DNA damage in rat alveolar macrophages and human monocyte cells.	(251)	The induction of micronuclei has been demonstrated following the dosage.	(252, 253)
Silica	The aforementioned factors result in cognitive dysfunction impairment, synaptic modifications, heightened oxidative stress, and alterations in microglial activity.	(254)	After a 24-hour exposure, it was observed that amorphous fumed silica caused considerable oxidative DNA damage in a human colon epithelial cell line. In a similar manner, the induction of genotoxicity was observed when human lymphoblastoid cells were exposed to ultrafine crystalline SiO_2_ nanoparticles with a size of 100 nm. The exposure durations were 6, 24, and 48 h, and a range of doses from 0 to 20 mg/ml was utilized.	(255, 256)	The inhalation of a recently produced aerosolized amorphous SiO_2_, with particle sizes of 37 and 82 nm, has been found to induce genotoxicity in rats. This genotoxic effect was observed within a brief timeframe of 1 to 3 days following exposure. The assessment of toxicity was conducted over a period ranging from 24 h to 2 months after the initial exposure.	(257)
Organic NPs: colloidal, polymeric solid lipid, vesicular, liposomes	The observed effects include inflammation, oxidative stress, neuronal death, and accumulation in the frontal brain, which are depending on the administered dose.	(258)	Dendrimers represent a class of highly branched polymers that have demonstrated considerable potential as a drug delivery system. The experimental findings revealed that the presence of cationic dendrimers led to a notable elevation in oxidative stress levels and DNA damage within human brain progenitor cells. This effect was observed to be contingent upon both the density of surface groups and the quantity of particles involved.	(259)	–	–

## 2 Summary

There are a lot of chances to modify and control the activity of cells and tissues at the nanoscale in the rapidly developing field of nanotechnology. The combination of bio- and nanotechnologies is transforming the approaches used to identify, treat, and track illnesses, thereby addressing both present-day and future medical issues. This issue showcases noteworthy advancements in the field of nanomedicine, which covers a wide range of medical issues such as tissue regeneration, dental health, cancer, tuberculosis, antibiotic resistance, and vaccination efficacy. NPs can, however, inadvertently cause harm to individuals even if they have advantageous qualities that make them very helpful in medical applications. Finding the precise physicochemical characteristics of NPs linked to toxicity is the main goal of the study of nanotoxicology, which ultimately aims to direct the creation of safe nanomaterials. Optimizing the safety profile of nanomaterials meant for use in medical contexts is the main goal of this field of study.
